# Multiplexed measurements of gene signatures in different analytes using the Nanostring nCounter™ Assay System

**DOI:** 10.1186/1756-0500-2-80

**Published:** 2009-05-09

**Authors:** Vladislav A Malkov, Kyle A Serikawa, Noel Balantac, James Watters, Gary Geiss, Afshin Mashadi-Hossein, Thomas Fare

**Affiliations:** 1 Rosetta Inpharmatics, LLC, 401 Terry Ave N, Seattle, WA 98109, USA; 2 Merck & Co Inc., West Point, PA 19486, USA; 3 NanoString Technologies, 530 Fairview Ave N, Seattle, WA 98109, USA; 4 Novo Nordisk, 530 Fairview Ave N, Seattle, WA 98109, USA

## Abstract

**Background:**

We assessed NanoString's nCounter™ Analysis System for its ability to quantify gene expression of forty-eight genes in a single reaction with 100 ng of total RNA or an equivalent amount of tissue lysate. In the nCounter™ System, multiplexed gene expression target levels are directly detected, without enzymatic reactions, via two sequence-specific probes. The individual mRNA is captured with one mRNA target sequence-specific capture probe that is used in a post-hybridization affinity purification procedure. The second mRNA target specific-sequence and fluorescent-labeled colored coded probe is then used in the detection with the 3-component complex separated on a surface via an applied electric field followed by imaging. We evaluated reproducibility, accuracy, concordance with quantitative RT-PCR, linearity, dynamic range, and the ability of the system to assay different inputs (matched samples of total RNA from Flash Frozen (FF) and Formalin Fixed Paraffin Embedded Tissues (FFPET), and crude tissue lysates (CTL)).

**Findings:**

The nCounter™ Analysis System provided data equivalent to that produced by Taqman^®^-based assays for genes expressed within the ranges of the calibration curves (above ~0.5 mRNA copies per human cell based on an assumption of 10 pg of total RNA per cell). System response was linear over more than two orders of magnitude with typical CVs of ~6% for concentrations above 1 fM (10^5 ^molecules per mL). Profiling the industry-standard MAQC data set yielded correlation coefficients of >0.83 for intensity values and >0.99 for measured ratios. Ninety percent of nCounter™ ratio measurements were within 1.27–1.33 fold changes of the Taqman^® ^data (0.34–0.41 in log_2 _scale) for FF total RNA samples.

**Conclusion:**

The nCounter™ Analysis System generated robust data for multi-gene expression signatures across three different sample preparation conditions.

## Background

Analyses of gene expression from microarrays can be used to define a specific set of sequences (signatures) relevant to a particular biological phenomenon or response [1]. These signatures can comprise tens to hundreds of genes, a range that falls between the optimal economic and logistic space for two widely-used tools for measuring gene expression, RT-PCR and microarrays. A solution for follow-up would provide cost-effective, multiplexed measurements of gene expression for tens to hundreds of genes while producing data equivalent to that generated by microarrays and RT-PCR. This solution should also be able to analyze input materials of clinical relevance (e.g., total RNA from formalin-fixed, paraffin embedded tissues (FFPET) and crude tissue lysates (CTL)).

As part of an internally funded investigation, Merck scientists tested the ability of the nCounter™ Analysis System [2] to meet this need via an experimental design (see Table 1) that featured both synthetic spike-in and "natural" total RNA for a set of 48 probes. Microarray Quality Consortium (MAQC) [3] total RNAs, total RNA from matched flash frozen (FF) and formalin fixed, paraffin embedded tissues, and CTL prepared from the same FF tissue were used as source materials for the assay. The work was contracted to Nanostring and the data analyzed at Merck.

**Table 1:** Experimental samples

Sample Type	# of Samples	Replicated?	Synthetic spike-ins added?	Total # assayed
MAQC samples (Brain, UHR, and 25%:75% proportional mixes of each)	4	Yes (1×)	Yes (?)	8

Tissue Lysates	10	No	Yes	10

Flash frozen total RNA (4 xenografts at 4 different dosage conditions)	16	Yes (1×)	Yes	32

FFPET total RNA	8	No	Yes	8

## Methods

### List of transcripts for the nCounter™ assay

The gene list for nCounter™ probes consisted of 14 human genes differentially regulated in our xenograft system, 25 human genes differentially regulated among the MAQC samples, and 9 sequences corresponding to synthetic transcripts used historically at Rosetta Inpharmatics as spike-ins for quality control (, Two-Color Microarray Spike-In Kit, part # 5188–5279, [4], Table 2). In addition, the nCounter™ Analysis System routinely includes spike-ins (cocktail #3 or #4,)[2]. These nCounter™ assay spike-in controls can be used for calibration and quality control purposes. For this work, the nCounter™ spike-in controls spanned 2.5 logs in concentration.

**Table 2:** Gene list for nCounter™ probe synthesis.

Gene/Gene Symbol	Representative Transcript/Transcript ID	Taqman probe ID (if used)
ASPA	NM_000049.2	

BCL6	NM_001706	Hs00153368_m1

C11orf58	NM_014267.3	

CCNG2	NM_004354	Hs00171119_m1

CDH1	NM_004360.2	

CHGB	NM_001819.1	

CUGBP1	NM_006560.2|NM_198700.1|NM_001025596.1	Hs00198069_m1

DNAJB9	NM_012328.1	

DYNLL1	NM_001037494.1|NM_001037495.1|NM_003746.2	

H6PD	NM_004285.3	Hs00188728_m1

HBEGF	NM_001945.1	

HIST1H1D	NM_005320.2	

HKR2	NM_181846.1	Hs00419189_m1

ICAM1	NM_000201.1	

IRS2	NM_003749	Hs00275843_s1

ITM2B	NM_021999.2	

LDHA	NM_005566.1	

LGI1	NM_005097.1	

MDS032	NM_018467.2	

MLLT7	NM_005938	Hs00172973_m1

MMP2	NM_004530.2	

MS4A6A	NM_152852.1|NM_022349.2|NM_152851.1	Hs00223521_m1

MXD4	NM_006454	Hs00170799_m1

MYC	NM_002467	Hs00153408_m1

NARG1	NM_057175	Hs00228208_m1

NIP7	NM_016101	Hs00602949_g1

NR0B2	NM_021969.1	

NTS	NM_006183.3	

PPARA	NM_001001928.2|NM_005036.4	

RNF10	NM_014868.3	

SEPT2	NM_001008491.1|NM_001008492.1|NM_006155.1|NM_004404.3	

SFRS10	NM_004593.1	

SHCBP1	NM_024745.2	

SLC25A32	NM_030780	Hs00229219_m1

TAF1A	NM_005681	Hs00375858_m1

TFRC	NM_003234.1	

THBS1	NM_003246.2	

TMSL8	NM_021992.2	

TP53	NM_000546.2	

r60_1	NA	

r60_3	NA	

r60_a104	NA	

r60_a107	NA	

r60_a135	NA	

r60_a20	NA	

r60_a22	NA	

r60_a97	NA	

r60_n11	NA	

r60_n9	NA	

### Samples

Samples (see Table 1) for the assay came from two sources: 1) EBC-1 lung cancer cell line xenograft tissues treated with vehicle or varying amounts of a compound; and 2) MAQC samples obtained from Ambion (Human Brain) and Stratagene (Universal Human Reference). Proportional mixes (25%:75% and 75%:25% UHR to Brain) were created. Crude tissue lysates (CTLs) were made by homogenizing 50–100 mg of FF xenograft tissues in 1 mL of Qiagen buffer RLT  and snap freezing a 100 μL aliquot (1/10^th ^of total volume). Total RNA was purified from the remaining lysate using a Trizol-based protocol . We isolated RNA from FFPE EBC1 xenografts using the Ambion RecoverAll protocol . In all samples, one or the other set of Rosetta spike-ins were added to provide a measure of ratio accuracy. Samples were blinded before assaying at Nanostring Technologies, where the samples were processed to generate raw data (i.e. counts/gene). nCounter™ assay spike-ins control mixes #3 or #4 were added at random to each of the blinded RNA samples on the day the assay was performed.

### The nCounter™ System assay

We performed the nCounter™ assay using 100 ng of total RNA or 2 μL of tissue lysate per replicate. Each assay was performed in triplicate to improve precision of the measurements. Details can be found in [2].

### RT-PCR

Fourteen genes were assayed via a Taqman^® ^quantitative RT-PCR protocol according to manufacturer's specifications using Applied Biosystem's High Capacity cDNA Reverse Transcription Kit (part # 4374967) and Taqman^® ^Universal PCR Master Mix (part # 4364340). Taqman reporter probes were used (see Table 1 for a list of specific ABI assay identifiers). An aliquot of 400 ng of total RNA was reverse-transcribed and 1/80^th ^of the reaction used for each replicate for each probe. The reaction volume for each replicate was 10 μL, with 0.5 μL of the Taqman 20× gene expression assay, 1 μL of sample, 5 μL of 2× Master Mix and 3.5 μL dH_2_O. All samples were assayed in quadruplicate for each probe according to Rosetta internal SOPs. Samples were run on an ABI 7900 HT system using the recommended ABI cycling protocol . See Table 2 for the specific Taqman^® ^identifiers for the 14 probes used.

### Data analysis

Eleven positive control nCounter™ spike-ins (spanning from 0.27 fM to 55 fM) were used to create the calibration curve for each nCounter™ array. Nine negative control spike-ins were used to assess the level of background (typically on the order of 10 counts). Mean of the negative controls was deducted from all other transcripts in the same assay prior to logarithmic transformation (log base 2). We used a standard linear regression model to find the least square fit of logarithm-transformed concentration on the logarithm-transformed number of molecules above background to generate the equation for the rest of the transcripts in the same assay. Each nCounter™ assay result was converted to an equivalent concentration using the assay standard curve. Use of the standard curve allows absolute measurements to be assigned to nCounter™ counts as needed.

To deduce the precision of the nCounter™ assay itself, we mean centered the data in log_2 _scale, resulting in a correction of approximately 1.08 fold. To achieve specified precision, NanoString recommends running each sample (by experiment) in triplicate. To mimic a typical experiment, therefore, we averaged triplicate assays for Rosetta spike-ins as well. Standard deviations of resulting mean values were used to calculate CVs.

To generate across-multiple-samples, gene-by-gene equivalency plots, both Taqman^® ^and nCounter™ data were normalized to CUGBP1 as a reference gene for the xenograft samples. Originally four reference genes were identified from previous experiments as not varying significantly across our experimental conditions and were planned to be used as references in aggregate. However, three of the four did not reliably give signals above the lower limit of our standard curve and so were not used. This led us to deviate from generally accepted practice in which more than one reference gene is used to normalize data.

In the MAQC analysis, although the published Taqman^® ^data were normalized to POLR2A, the nCounter™ normalization did not utilize any reference genes. This is because POLR2A was not one of the genes present in our genelist and so was unavailable as a common control. As a result of this approach, Taqman^® ^data were normalized to mRNA amount, while nCounter™ normalization relied on the same amount of total RNA (100 ng) in each sample. This distinction is important because the MAQC study showed that UHR has 1.5-fold higher mRNA content than Brain (3% vs. 2%). To compensate for different mRNA content, 0.585 Ct, 0.46 Ct and 0.17 Ct were deducted from all genes of 0% Brain/100% UHR; 25% Brain/75% UHR; and 75% Brian/25% UHR samples, respectively.

For performance evaluations, a comparison was done for each possible pair of samples because we did not wish to artificially bias our data by arbitrarily assigning one sample as the "standard" to which other replicates would be compared. In these cases, we normalized to the mean of log intensity of the subset of genes in the corresponding sample for which measurements were above 0.27 fM in both samples of the pair. The same subset of genes was used to normalize Taqman^®^, using their mean Ct.

Assessment of the Spike-in performance and of the MAQC samples can be found in Additional File 1, Additional File 2, Additional File 3, and Additional File 4, and in Tables 3 and 4. Data from the analysis can be found in Additional File 5.

**Table 3:** Expected and back-calculated (observed) concentrations in fmoles of Nanostring spike-in mixes 3 and 4, including %CV and %Bias.

	Nanostring spike-in Mix #3					Nanostring spike-in Mix #4				
Spike Name	Expected	Observed	StdDwev	CV, %	Bias, %	Expected	Observed	StdDev	CV, %	Bias, %

S23	50	61.45	3.48	5.7	22.9	50	59.45	3.63	6.1	18.9

S14	5	3.36	0.28	8.5	32.9	5	3.24	0.31	9.5	35.2

S19	0.5	0.42	0.07	17.2	16.5	0.5	0.45	0.08	17.3	10.6

S8	5	3.19	0.24	7.5	36.2	15	9.94	0.62	6.3	33.7

S13	15	10.74	0.48	4.5	28.4	5	3.08	0.31	10.0	38.5

S22	5	4.72	0.30	6.3	5.5	50	56.19	3.77	6.7	12.4

S7	50	53.23	2.67	5.0	6.5	5	4.36	0.30	7.0	12.9

S17	1.5	2.41	0.25	10.4	60.8	4.5	7.96	0.75	9.4	76.9

S3	4.5	5.4	0.33	6.0	19.9	1.5	1.62	0.19	11.8	7.8

S6	0.25	0.31	0.07	21.8	23.3	0.75	1.02	0.13	12.8	35.7

S4	0.75	1.06	0.13	12.1	41.5	0.25	0.38	0.07	17.7	50.4

Calculations based on all replicates.

**Table 4:** Expected and observed concentrations for Rosetta spike-ins 11 and 12, including %CV and %Bias.

Rosetta spike-in #11						
Transcript name	Intended conc, AU	Best Fit Expected	Observed	StdDev	CV, %	Bias, %

r60_a20:50:rp	100	32.09	28.62	1.47	5.2	10.8

r60_a22_rp	10	3.21	4.71	0.28	5.9	46.9

r60_a104_rp	10	3.21	2.62	0.14	5.2	18.5

r60_1_rp	10	3.21	4.29	0.26	6.1	33.6

r60_a107_rp	30	9.63	10.56	0.70	6.6	9.7

r60_3_rp	3	0.96	1.02	0.09	9.1	6.1

r60_a135_rp	9	2.89	3.13	0.16	5.0	8.3

r60_a97_rp	0.5	0.16	0.14	0.03	21.8	15.5

r60_n11:30:rp	1.5	0.48	0.34	0.07	21.3	30.1

Rosetta spike-in #12						

r60_a20:50:rp	100	44.18	37.56	2.25	6.0	15.0

r60_a22_rp	100	44.18	81.54	5.51	6.8	84.5

r60_a104_rp	30	13.25	12.16	0.70	5.8	8.2

r60_1_rp	10	4.42	5.85	0.30	5.1	32.3

r60_a107_rp	10	4.42	4.49	0.36	8.0	1.6

r60_3_rp	9	3.98	4.28	0.25	5.8	7.7

r60_a135_rp	3	1.33	1.30	0.08	6.2	1.6

r60_a97_rp	1.5	0.66	0.61	0.07	11.3	7.9

r60_n11:30:rp	0.5	0.22	0.12	0.04	29.7	43.6

Calculations based on a per-sample basis.

## Results and discussion

### Analysis of Xenograft-derived Samples

The sample set comprised four treatment conditions with four mouse xenografts per condition for a total of 16 samples. The 16 tissue samples were split and preserved by three methods (FF, FFPET, and CTL in Qiagen buffer RLT); total RNA was isolated from FF and FFPET for RT-PCR analysis. Fourteen genes were chosen for Taqman^® ^comparison using samples that were either vehicle treated or treated with the highest level of compound. Ten genes were expected to change either up or down, and four reference genes were expected to remain constant. The genes chosen for Taqman^® ^and Nanostring comparisons were picked based on internal Merck criteria. The differential expression in a previous microarray study of these samples showed relatively modest fold changes (~2 fold) at the highest compound treatment level used for this study (data for other, intermediate treatment levels is not shown).

**Figure 1:**
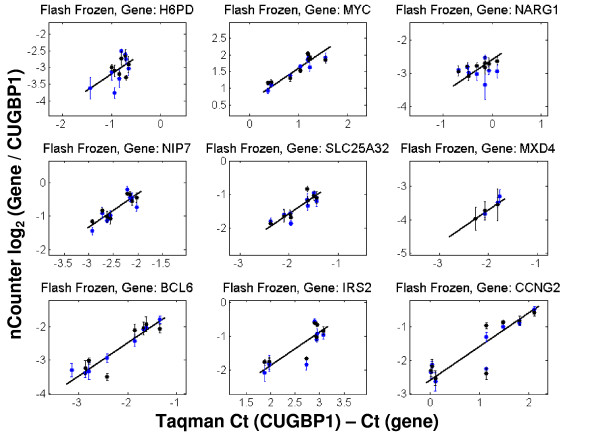
**Comparison of intensities derived from Taqman^® ^and nCounter™ for FF-derived total RNA**. The line in each case represents a slope of 1. Blue and black dots represent the two replicate measurements for each mouse sample. Only data points within the calibration curve are presented in these graphs. Units for the x-axis are delta CTs using CUGBP1 to normalize; units for the y-axis are log_2 _ratios of nCounter™ counts for a given gene and CUGPB1. Error bars represent standard deviations.

**Figure 2:**
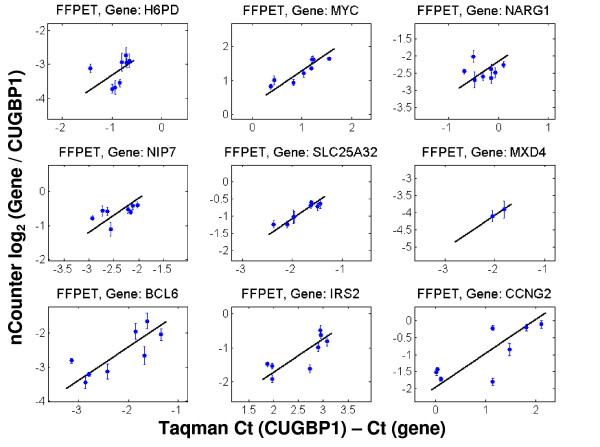
**Comparison of intensities derived from Taqman^® ^on FF total RNA and nCounter™ on FFPET-derived total RNA**. The line in each case represents a slope of 1. Only data points within the calibration curve are presented in these graphs. Units for the x-axis are delta CTs using CUGBP1 to normalize; units for the y-axis are log_2 _ratios of nCounter™ counts for a given gene and CUGPB1. Error bars represent standard deviations.

**Figure 3:**
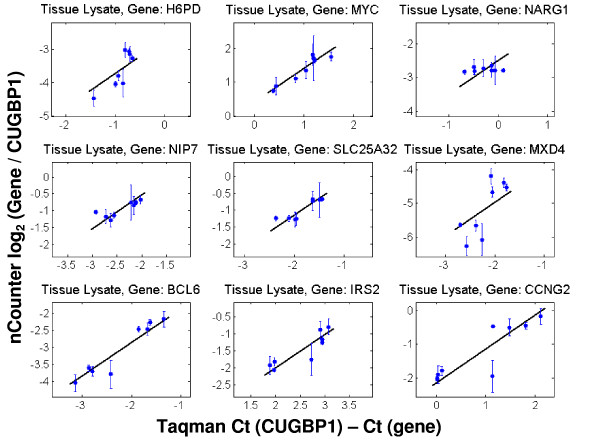
**Comparison of intensities derived from Taqman^® ^on FF total RNA and nCounter™ on tissue lysates**. The line in each case represents a slope of 1. Only data points within the calibration curve are presented in these graphs. Units for the x-axis are delta CTs using CUGBP1 to normalize; units for the y-axis are log_2 _ratios of nCounter™ counts for a given gene and CUGPB1. Error bars represent standard deviations.

A comparison of the compound and vehicle intensities between nCounter™ and Taqman^® ^is shown in Figures 1, 2 and 3 for nine of the ten genes expected to change between treatments. Reference genes are not shown and one of the ten differentially expressed genes was not present at a sufficiently high level for accurate assessment. Figure 1 corresponds to FF tissue; Figure 2 to FFPET; and Figure 3 to CTL. In Figures 1, 2 and 3 only those points with expression levels within the calibration range are shown. The reference gene CUGBP1 was used to calculate the delta-delta CT for all genes.

As with the MAQC data, these sample sets show generally good agreement between the two platforms. The one outlier is NARG1 (top right graph in each Figure), which was consistently discordant in all Taqman^® ^to nCounter™ comparisons. Since the region of NARG1 assayed by the Taqman^® ^probe is at the junction between exons 1 and 2 and the region selected for the nCounter™ probe is close to the 3' end of the transcript, the two systems may be capturing valid but different transcript behavior of this gene. It should be noted that variability of measurements in the CTL samples (as represented by the error bars) was higher than for the other two sample types.

The FF and lysate data have a high degree of correlation, suggesting minor loss of data quality by using CTLs rather than purified total RNA. Taken together, the data in Figures 1, 2 and 3 suggest that the nCounter™ assay can be used to generate data from clinical samples with degraded RNA (FFPET, see Additional File 6 for representative quality) or from lysate preparations. It should be pointed out, however, that the FFPET model used (xenograft tissue) is not a perfect match for typical clinical samples and that not all degrees of degraded RNA will be amenable to this system

### Performance metrics

**Figure 4:**
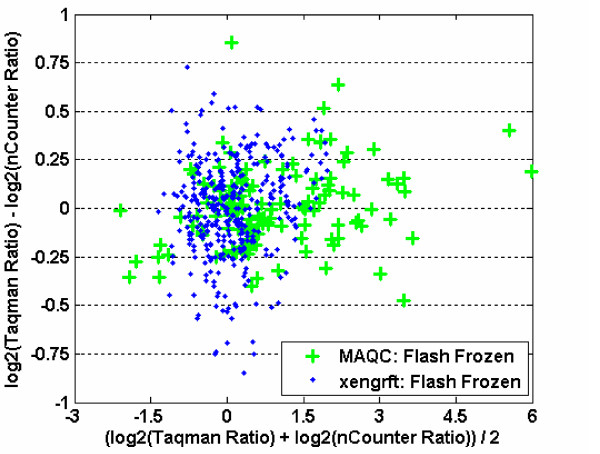
**Plot for ratio differences between nCounter™ and Taqman^®^**. The differences in ratio between Taqman^® ^and nCounter™ for the MAQC and xenograft data sets were plotted versus average ratio of two platforms. Markers in blue represent ratios derived for xenograft samples; markers in green represent ratios derived for MAQC samples.

To assess bias or compression in derived ratios, we plotted differences in ratios between nCounter™ and Taqman^® ^versus average of ratios reported by both platforms (Figure 4). The scatter around the abscissa axis (y = 0) of *differences *suggests no bias in the measurement of ratios by nCounter™ relative to Taqman^®^, while the random scatter across *degrees of differential expression *(along the x-axis)implies no compression of ratios across ratio values.

**Figure 5:**
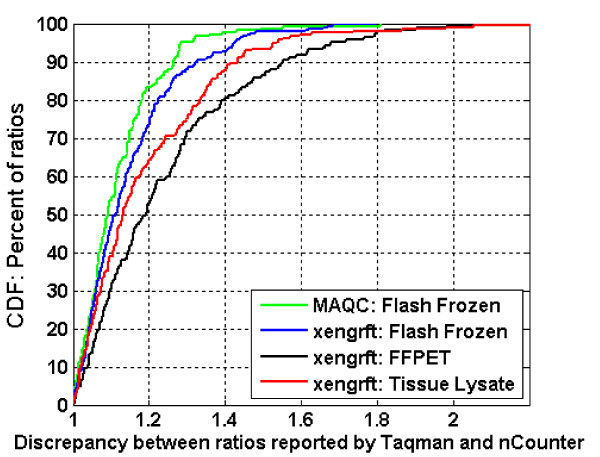
**Cumulative distribution function plots for difference between Taqman^® ^and nCounter™ derived ratios**. Values on the x-axis are the absolute value of the difference between Taqman^® ^and nCounter™ in log_2 _scale. The y-axis indicates the percentage of ratios which show a specific degree of difference between ratios or less.

Figure 5 displays a set of cumulative distribution function (CDF) curves for ratios reported by the nCounter™ assay for the MAQC and the three xenograft datasets (FF, FFPET and CTL) versus the Taqman^®^-derived ratios from the FF samples. The absence of ratio compression simplifies the subsequent analysis: since the magnitude of the ratio does not affect the concordance between two platforms by this method, we can combine all measurements together and determine how many measurements were off and by how much. Rather than depend solely on one references gene, we performed a comparison of all possible sample pairs; systematic shifts affecting all genes in each comparison were thereby attributed to the difference in total mass and subtracted out. As expected, ratios measured by nCounter™ and Taqman^® ^were similar for MAQC and FF xenograft samples. The MAQC samples showed slightly less deviation between Taqman^® ^and the nCounter™ System, which may reflect the controlled nature of the sample set and the smaller fraction of very-low intensity genes (see Table 3 for CV dependence on transcripts abundance). Overall the differences are small–for example, 90% of all nCounter™ measurements were within 1.27 and 1.33 fold changes of the Taqman^® ^calculated ratio for the MAQC and cell line FF data, respectively. Interestingly, nCounter™ data from the CTL samples more closely mirrored the Taqman^® ^data than did the FFPET total RNA data. However, even ratios reported by nCounter™ FFPET samples are close to Taqman^® ^ratios obtained for FF samples: 90% of ratios were within 1.55 fold.

Our impetus to assess the nCounter™ Analysis System was driven by its relative simplicity (that is, no need for amplification steps), its multiplexed format, and its potential to measure gene expression in samples from pre-clinical and clinical settings (e.g, fine-needle biopsies in lysate buffers, and FFPET materials). Our results confirmed the system has potential for pre-clinical and clinical measurements of multiple gene signatures in settings where the initial tissue collection would be conducive to FFPET or CTL preparations.

This platform could be used to fill an important and growing gap in drug development research. Microarray experiments routinely are used in basic research but often identify too many genes to allow higher-throughput downstream use of those signatures for screening or readouts. By allowing the cost effective and accurate measurement of expression of tens of genes from clinical samples, the nCounter™ system could facilitate translation of multi-gene expression based biomarkers into the clinic.

## Competing interests

GG and AM-H are employees of Nanostring.

## Authors' contributions

VM performed the data transformations and analysis; KS designed and initiated the experiments and assembled the sample set, and also was the primary author of the manuscript; NB performed the Taqman assays; JW provided the xenograft model system and the tissue samples used, and also provided critical review of experimental design; GG and AM-H processed the samples at Nanostring and provided technical and critical review of the manuscript; TF provided overall project guidance, technical and critical review of the experiments and assisted in writing. All authors read and approved the final manuscript.

## Supplementary Material

Additional file 1 **Performance of Nanostring spike-ins**. Nanostring spike-ins span a reported range from 0.25 fM to 50 fM. All samples were assayed in triplicate; data are broken down by replicate. nCounter™ spike-ins consisted of two parallel sets of the same RNAs made into two different cocktails (cocktails #3 and #4). Some RNAs are set at different concentrations between these cocktails while others are at the same concentration (Table 3). Click here for file

Additional file 2 **Performance of Rosetta spike-ins**. The Rosetta spike-ins were originally constructed to measure dynamic range, ratio fidelity, and hybridization quality of an in-house, two-color microarray system [4]. Data from the nCounter™ spike-in controls were used to derive the precision profile and to provide a calibration curve for quantitation of other RNA transcripts. Spike-ins curves showed good linearity and reproducibility, and good conservation of relative measurements. Overall, the precision of the platform for the Rosetta Spike-ins had CVs of approximately 6%, except for concentrations below 1.5 fM (CVs were 10–30%). Error bars represent standard deviations. Click here for file

Additional file 3 **Comparison of intensity values derived from Taqman^® ^(x-axis) and Nanostring (y-axis) for each of the four MAQC samples (UHR, 25% Brain/75% UHR, 75% Brain/25% UHR, and Brain)**. Markers in red represent genes that were outside of the calibration curve. Pair-wise nCounter™ to Taqman^® ^comparisons of measured expression levels of genes on a per sample basis showed correlation coefficients ranging from 0.835 to 0.886. Error bars represent standard deviations. Click here for file

Additional file 4 **Comparison of calculated log_2 _ratios for Brain vs UHR for Nanostring and Taqman^®^**. Transcripts that were outside the calibration curve are shown in red and are labeled with their lowest measured concentration in either Brain or UHR (in fM). In the comparison of ratios, data for nCounter™ and Taqman^® ^were first corrected for the known differences in mRNA content between Brain and UHR (2% to 3%). Additional File 4 shows the fit of the ratio comparisons to the line y = x for samples whose intensity falls within the standard curve (blue squares in Additional File 4), R = 0.995, demonstrating good linear agreement between Taqman^® ^and nCounter™ ratio measurements. Click here for file

Additional file 5 **Data from Nanostring nCounter experiment**. This spreadsheet contains the raw nCounter™ data, the Taqman^® ^data used for comparisons, and the normalized and averaged nCounter™ data. Click here for file

Additional file 6 **Representative Bioanalyzer traces from FFPE-derived RNA**. These traces demonstrate the degree of RNA degradation assayed by the nCounter™ system in our experiments. Click here for file

## References

[B1] Van't Veer LJ, Dai HY, Vijver MJ van de, He YDD, Hart AAM, Mao M, Peterse HL, Kooy K van der, Marton MJ, Witteveen AT, Schreiber GJ, Kerkhoven RM, Roberts C, Linsley PS, Bernards R, Friend SH (2002). Gene expression profiling predicts clinical outcome of breast cancer. Nature.

[B2] Geiss GK, Bumgarner RE, Birditt B, Dahl T, Dowidar N, Dunaway DL, Fell HP, Ferree S, George RD, Grogan T, James JJ, Maysuria M, Mitton JD, Oliveri P, Osborne JL, Peng T, Ratcliffe AL, Webster PJ, Davidson EH, Hood L, Dimitrov K (2007). Direct multiplexed measurement of gene expression with color-coded probe pairs. Nature Biotechnology.

[B3] Shi L, Reid LH, Jones WD, Shippy R, Warrington JA, Baker SC, Collins PJ, de Longueville F, Kawasaki ES, Lee KY, Luo Y, Sun YA, Willey JC, Setterquist RA, Fischer GM, Tong W, Dragan YP, Dix DJ, Frueh FW, Goodsaid FM, Herman D, Jensen RV, Johnson CD, Lobenhofer EK, Puri RK, Schrf U, Thierry-Mieg J, Wang C, Wilson M, Wolber PK, Zhang L, Amur S, Bao W, Barbacioru CC, Lucas AB, Bertholet V, Boysen C, Bromley B, Brown D, Brunner A, Canales R, Cao XM, Cebula TA, Chen JJ, Cheng J, Chu TM, Chudin E, Corson J, Corton JC, Croner LJ, Davies C, Davison TS, Delenstarr G, Deng X, Dorris D, Eklund AC, Fan XH, Fang H, Fulmer-Smentek S, Fuscoe JC, Gallagher K, Ge W, Guo L, Guo X, Hager J, Haje PK, Han J, Han T, Harbottle HC, Harris SC, Hatchwell E, Hauser CA, Hester S, Hong H, Hurban P, Jackson SA, Ji H, Knight CR, Kuo WP, LeClerc JE, Levy S, Li QZ, Liu C, Liu Y, Lombardi MJ, Ma Y, Magnuson SR, Maqsodi B, McDaniel T, Mei N, Myklebost O, Ning B, Novoradovskaya N, Orr MS, Osborn TW, Papallo A, Patterson TA, Perkins RG, Peters EH, Peterson R, Philips KL, Pine PS, Pusztai L, Qian F, Ren H, Rosen M, Rosenzweig BA, Samaha RR, Schena M, Schroth GP, Shchegrova S, Smith DD, Staedtler F, Su Z, Sun H, Szallasi Z, Tezak Z, Thierry-Mieg D, Thompson KL, Tikhonova I, Turpaz Y, Vallanat B, Van C, Walker SJ, Wang SJ, Wang Y, Wolfinger R, Wong A, Wu J, Xiao C, Xie Q, Xu J, Yang W, Zhang L, Zhong S, Zong Y, Slikker W, MAQC Consortium (2006). The MicroArray Quality Control (MAQC) project shows inter- and intraplatform reproducibility of gene expression measurements. Nat Biotechnol.

[B4] Hughes TR, Mao M, Jones AR, Burchard J, Marton MJ, Shannon KW, Lefkowitz SM, Ziman M, Schelter JM, Meyer MR, Kobayashi S, Davis C, Dai H, He YD, Stephaniants SB, Cavet G, Walker WL, West A, Coffey E, Shoemaker DD, Stoughton R, Blanchard AP, Friend SH, Linsley PS (2001). Expression profiling using microarrays fabricated by an ink-jet oligonucleotide synthesizer. Nat Biotechnol.

