# Supplementary figures and images for: Multiplexed measurements of gene signatures in different analytes using the Nanostring nCounter™ Assay System
Source: BMC Res Notes. 2009 May 9;2:80. doi: 10.1186/1756-0500-2-80 (PMC2688518; doi:10.1186/1756-0500-2-80)

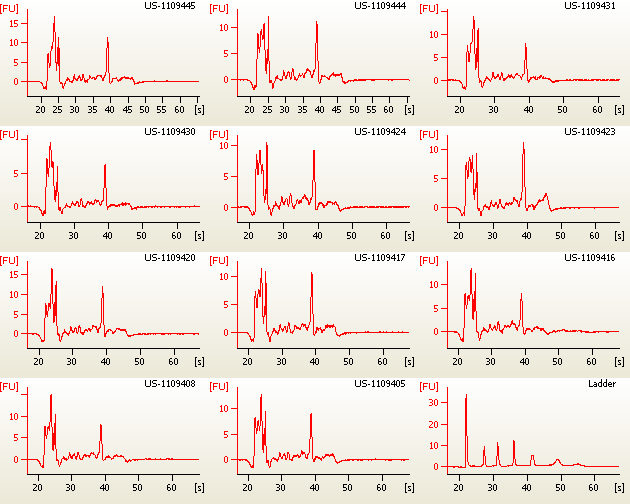

Supplement: Additional file 6 — Representative Bioanalyzer traces from FFPE-derived RNA. These traces demonstrate the degree of RNA degradation assayed by the nCounter™ system in our experiments. [file 1756-0500-2-80-S6.doc]
