# Supplementary material for: Multiplexed measurements of gene signatures in different analytes using the Nanostring nCounter™ Assay System
Source: BMC Res Notes. 2009 May 9;2:80. doi: 10.1186/1756-0500-2-80 (PMC2688518; doi:10.1186/1756-0500-2-80)
Supplement: Supplementary file 3 [file 1756-0500-2-80-S1.doc]

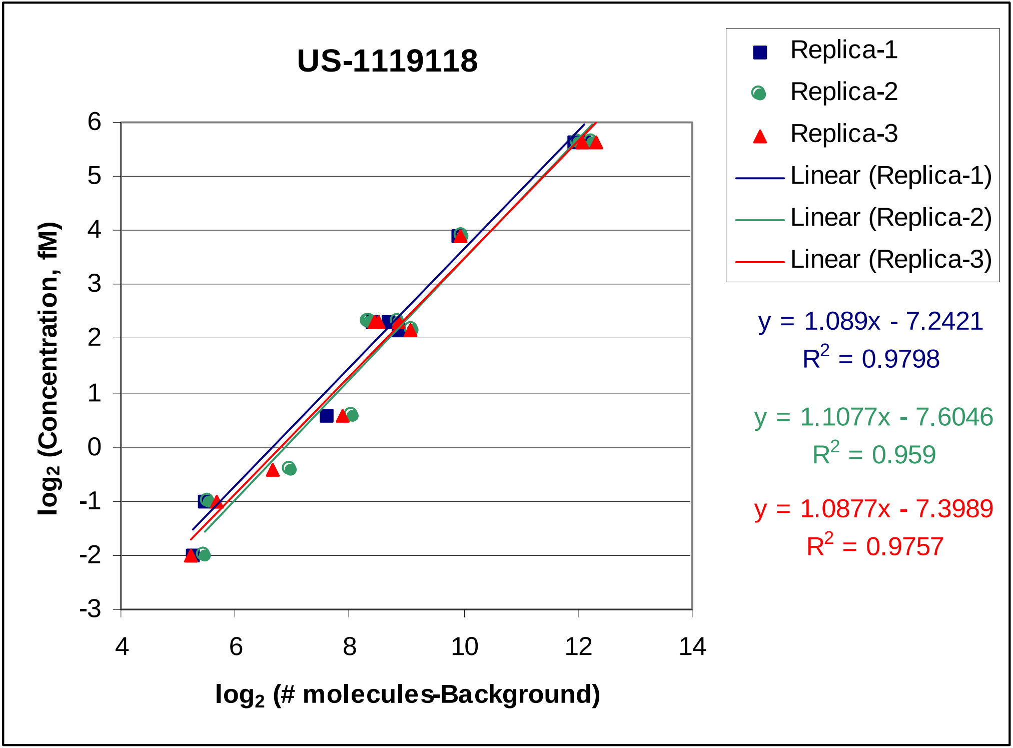
US-1119118
6
5
4
3
2
1
0
-1
-2
-3
4
6
8
10
12
14
log2 (Concentration, fM)
log2 (# molecules-Background)
Replica-1
Replica-2
Replica-3
Linear (Replica-1)
Linear (Replica-2)
Linear (Replica-3)
y = 1.089x - 7.2421
R2 = 0.9798
y = 1.1077x - 7.6046
R2 = 0.959
y = 1.0877x - 7.3989
R2 = 0.9757
