# Supplementary material for: Multiplexed measurements of gene signatures in different analytes using the Nanostring nCounter™ Assay System
Source: BMC Res Notes. 2009 May 9;2:80. doi: 10.1186/1756-0500-2-80 (PMC2688518; doi:10.1186/1756-0500-2-80)
Supplement: Supplementary file 4 [file 1756-0500-2-80-S2.doc]

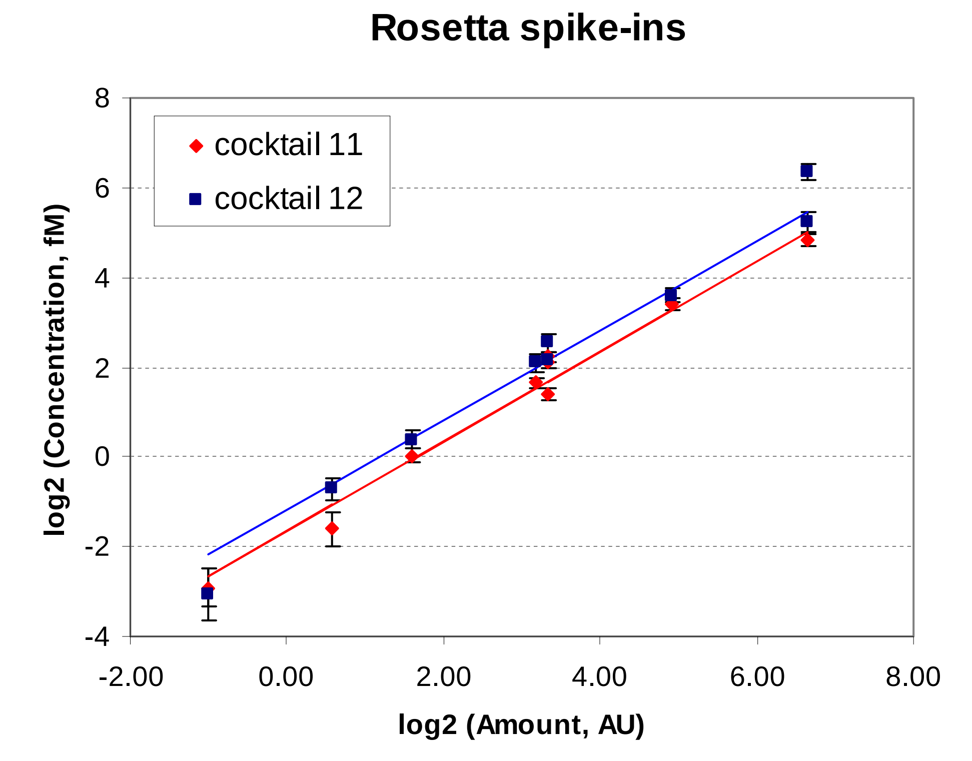

Rosetta spike-ins
cocktail 11
cocktail 12
8
6
4
2
0
-2
-4
-2.00
0.00
2.00
4.00
6.00
8.00
log2 (Concentration, fM)
log2 (Amount, AU)
