# Supplementary material for: Multiplexed measurements of gene signatures in different analytes using the Nanostring nCounter™ Assay System
Source: BMC Res Notes. 2009 May 9;2:80. doi: 10.1186/1756-0500-2-80 (PMC2688518; doi:10.1186/1756-0500-2-80)
Supplement: Supplementary file 5 [file 1756-0500-2-80-S3.doc]

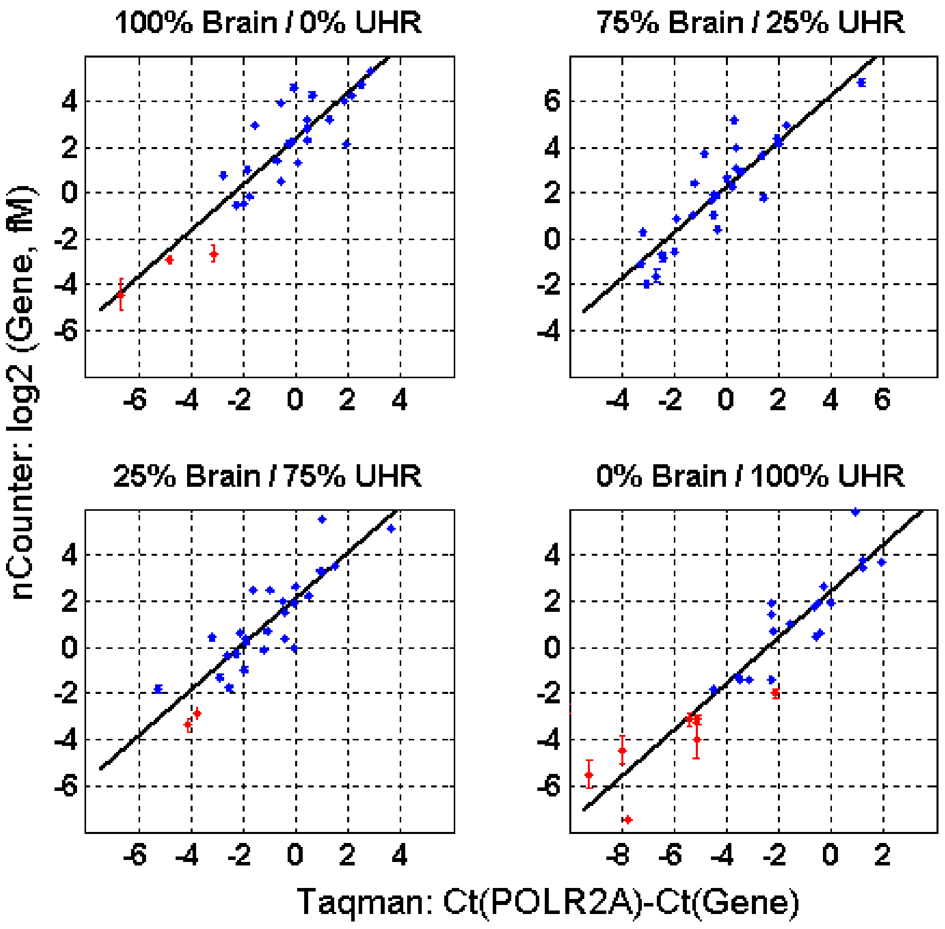

100% Brain / 0% UHR
75% Brain / 25% UHR
25% Brain / 75% UHR
0% Brain / 100% UHR
nCounter: log2 (Gene, fM)
Taqman: Ct(POLR2A)-Ct(Gene)
