# Supplementary material for: Multiplexed measurements of gene signatures in different analytes using the Nanostring nCounter™ Assay System
Source: BMC Res Notes. 2009 May 9;2:80. doi: 10.1186/1756-0500-2-80 (PMC2688518; doi:10.1186/1756-0500-2-80)
Supplement: Supplementary file 6 [file 1756-0500-2-80-S4.doc]

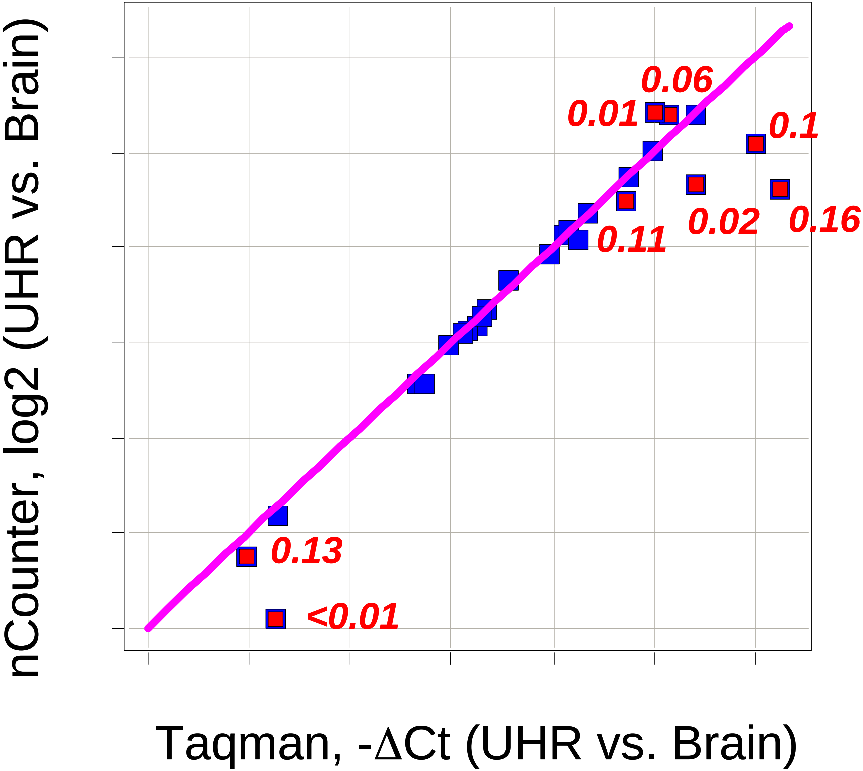

nCounter, log2 (UHR vs. Brain)
Taqman, -ΔCt (UHR vs. Brain)
0.06
0.01
0.1
0.02
0.16
0.11
0.13
<0.01
